# pIgR Stem Zone-Targeted Nanobodies as Apical-to-Basolateral Carriers for Inhaled Biologic Delivery Across Mucosal Barriers

**DOI:** 10.3390/antib15040053

**Published:** 2026-06-23

**Authors:** Aidong Qiu, Ruiyuan Wang, Yangyingjie Bai, Bowen Zhang, Xinyu He, Jiani Xie, Jianghai Liu

**Affiliations:** 1 College of Food and Bioengineering, Chengdu University, Chengdu 610106, China; wangruiyuan0212@163.com (R.W.); byyjxxs@163.com (Y.B.); xiejiani@cdu.edu.cn (J.X.); 2 Ablink Biotech, Chengdu 610200, China; bowen.zhang@ablinkbiotech.com (B.Z.); 18980347515@163.com (X.H.); 3 School of Bioscience and Technology, Chengdu Medical College, Chengdu 610500, China

**Keywords:** mucosal barrier, pIgR, nanobody, transepithelial transport, apical-to-basolateral, asthma

## Abstract

Background: The mucosal barrier presents a significant challenge for non-invasive delivery of macromolecular therapeutics, often requiring administration with poor bioavailability and increased toxicity risks. The polymeric immunoglobulin receptor (pIgR) contains an extracellular secretory component (SC) for immunoglobulin binding and a membrane-anchored stem domain capable of apical-to-basolateral transcytosis. We hypothesized that targeting the stem domain could enable active drug transport across mucosal barriers. Methods: Using phage display, we identified four high-affinity nanobodies against human and murine pIgR. Two lead candidates (3LTHMP-4 and 3LTHMP-5) demonstrated efficient apical-to-basolateral transport in vitro (Transwell assays) and in vivo (fluorescence imaging). Engineered bispecific antibodies fusing these nanobodies with anti-IL-5 mAb reslizumab were administered via inhalation in a murine asthma model at one-tenth the intraperitoneal reslizumab dose. Resluts: The bispecific antibodies showed significant therapeutic efficacy, while reslizumab alone at equivalent concentrations failed to demonstrate efficacy. Hydrogen–Deuterium Exchange Mass Spectrometry (HDX-MS) revealed that both 3LTHMP-4 and 3LTHMP-5 specifically bind to the pIgR stem domain (residues 578–612), a region distinct from the dimeric IgA binding site. Conclusions: These findings suggest that stem domain-specific binding may facilitate transport across the mucosal barrier while preserving native receptor physiology, offering a potential strategy for effective transmucosal delivery of biologics.

## 1. Introduction

The mucosal surface, lining the respiratory, gastrointestinal, reproductive, and ocular tracts, is the body’s primary interface with the external environment [1]. While these epithelial barriers are essential for host defense [2], they also present formidable challenges for drug delivery [3]. Large-molecule therapeutics, such as monoclonal antibodies, are often highly effective for disease [4]; however, their size and hydrophilicity inherently prevent them from crossing the mucosal membrane. As a result, nearly all biologic drugs must be administered via injection, which compromises patient convenience and treatment adherence. A platform technology capable of actively traversing the mucosal barrier to deliver biologic agents could fundamentally transform drug administration for a wide range of diseases.

**Figure 1:**
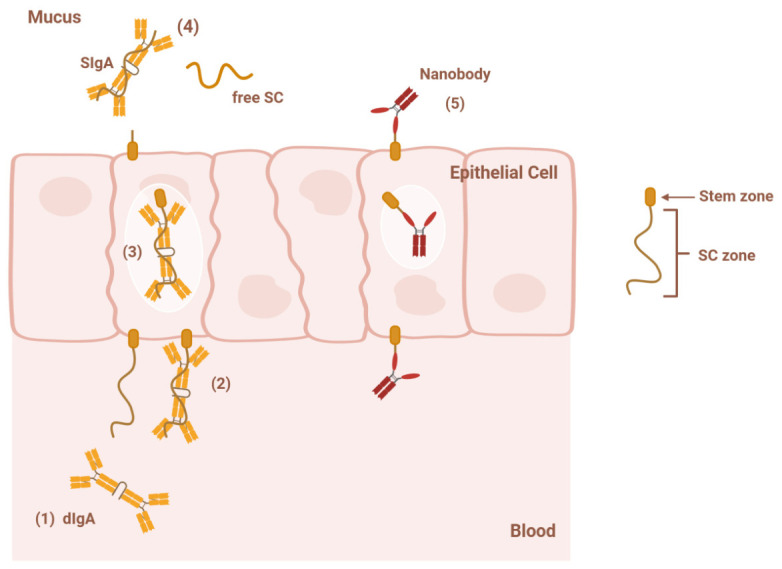
The pIgR-mediated transport mechanism: (1) Immune cells secrete dIgA. (2) dIgA binds pIgR at the basolateral membrane. (3) The complex undergoes transcytosis. (4) At the apical surface, proteolytic cleavage releases secretory IgA (SIgA) into mucus while the pIgR stem region remains membrane-associated. (5) Nanobodies targeting the pIgR stem zone mediate apical-to-basolateral transcytosis [17].

To address these challenges, the polymeric immunoglobulin receptor (pIgR) emerges as a particularly promising target. This glycosylated type I transmembrane protein, predominantly expressed on epithelial cells [5], mediates transcellular transport of immunoglobulin A (IgA) and M (IgM) across epithelial barriers, playing a pivotal role in mucosal and innate immunity [6]. The pIgR consists of two structurally and functionally distinct domains: (1) the extracellular secretory component (SC) that binds and transports polymeric immunoglobulins (dimeric IgA or pentameric IgM) at the basolateral membrane, and (2) a membrane-proximal stem region (amino acids 566–638) that anchors the receptor. Following ligand binding at the basolateral surface, the pIgR–immunoglobulin complex undergoes endocytosis and transcytosis to the apical membrane [5]. At the apical surface, proteolytic cleavage releases the SC-bound immunoglobulin complex (forming secretory IgA or IgM) into the mucosal lumen, where it participates in immune defense [7]. Concurrently, the membrane-retained stem region can undergo apical-to-basolateral transport back to the basolateral membrane [8], completing a transcytosis-recycling cycle (Figure 1). This raises the intriguing possibility that antibodies targeting the stem region could exploit this apical-to-basolateral pathway to be actively delivered from the mucosal surface into the underlying tissue. This transcytosis-recycling mechanism, combined with pIgR’s pan-mucosal distribution [9,10,11], establishes a versatile platform for non-invasive biologic delivery across multiple tissue types.

In this study, we demonstrate the therapeutic potential of our pIgR-targeted delivery platform for respiratory diseases using asthma as a model system—a global health challenge affecting over 300 million individuals, about 10% of whom suffer from severe, treatment-resistant forms [12,13,14,15]. While biologics like anti-IL-5 antibodies (e.g., reslizumab) have shown clinical efficacy, their injectable administration and poor mucosal permeability remain significant limitations [3,16]. Using phage display technology, we identified two lead nanobody candidates (3LTHMP-4 and 3LTHMP-5) that specifically target the pIgR stem region. These nanobodies demonstrated high cross-species affinity and efficient apical-to-basolateral transcytosis capability in polarized epithelial cell models. When engineered as bispecific antibody constructs by fusing with anti-IL-5 monoclonal antibody reslizumab and administered via inhalation in a murine asthma model, when delivered intratracheally at one-tenth the intraperitoneal administration dose, the bispecific antibodies demonstrated markedly superior therapeutic efficacy to Reslizumab alone. These results position pIgR-targeted nanobodies as a versatile platform technology for non-invasive biologic delivery, with demonstrated efficacy in respiratory applications and potential applicability across multiple mucosal surfaces.

## 2. Materials and Methods

### 2.1. Isolation of pIgR-Specific Nanobodies

**Figure 2:**
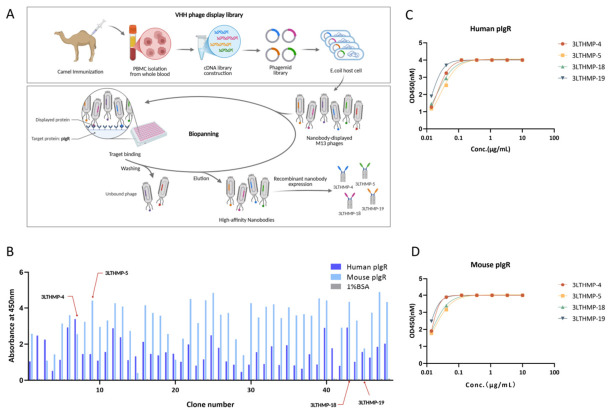
Identification and characterization of pIgR-specific nanobodies. (**A**) Schematic overview of the phage display selection process used to isolate pIgR-specific nanobodies from a camelid immune library. (**B**) Phage ELISA of 48 randomly selected clones showing binding reactivity to human pIgR, mouse pIgR, and 1%BSA (negative control). (**C**,**D**) Binding affinity (EC_50_) of purified nanobody-Fc fusion proteins to (**C**) human pIgR and (**D**) mouse pIgR, as determined by ELISA.

To generate nanobodies recognizing both human and murine pIgR, a camel was immunized with recombinant extracellular domains (ECD) of human and mouse pIgR (Ablink Biotech, Chengdu, China). Following the third booster immunization, peripheral blood was collected, peripheral blood mononuclear cells (PBMC) were isolated, and total RNA was extracted. VHH sequences were amplified by a two-step nested PCR using specific primers, and the resulting amplicons were cloned into a phagemid vector to construct a nanobody library for subsequent selection [18]. pIgR-specific nanobodies were isolated through successive rounds of phage display biopanning. Briefly, phage particles displaying the nanobody library were incubated with immobilized pIgR-ECD. Unbound phages were removed by washing, and specifically bound phages were eluted, amplified, and subjected to three additional rounds of panning under increasingly stringent conditions. After the fourth round, individual clones were picked, and 48 randomly selected clones were screened for antigen binding using a standard protocol [19] (Figure 2A).

### 2.2. Phage ELISA

The 48 clones selected after the fourth round of biopanning were inoculated into a 96-well deep-well plate containing 600 μL of 2YT medium (Starmetre, Shanghai, China) supplemented with 100 μg/mL carbenicillin and 25 μg/mL kanamycin, and incubated at 37 °C for 20 h. The plate was then centrifuged at 3000× *g* for 10 min, and the phage-containing supernatant was collected for ELISA. For antigen coating, 96-well plates were coated overnight at 4 °C with 100 μL per well of either human or mouse pIgR-ECD (1 μg/mL in PBS) or 1% bovine serum albumin (BSA; Sigma, St. Louis, MO, USA) as a negative control. After blocking with 1% polyvinyl alcohol (PVA; Sigma) for 2 h at room temperature (RT), the plates were washed three times with PBS (Biosharp, Beijing, China). Phage supernatant (100 μL per well) was added and incubated for 2 h at RT. Following three washes with PBS, horseradish peroxidase (HRP)-conjugated anti-M13 antibody (SB), diluted 1:5000, was added and incubated for 1 h at RT. The plates were then washed three times with PBST and three times with PBS. Color development was performed using 3,3′,5,5′-tetramethylbenzidine (TMB) substrate (Solarbio, Beijing, China), and the reaction was stopped by adding 1 M H_3_PO_4_. Absorbance at 450 nm was measured using a microplate reader (Thermo Fisher Scientific, Waltham, MA, USA). Clones exhibiting an absorbance value at least five-fold above the BSA control were considered positive and selected for further characterization.

### 2.3. Expression and Purification of Nanobody-Fc Fusions

The coding sequences of selected nanobody clones were subcloned into mammalian expression vectors encoding a C-terminal human IgG1 Fc tag. The resulting plasmids were transfected into suspension-adapted Expi293F cells (Thermo Fisher Scientific, Waltham, MA, USA) using FectoPRO^®^ (Polyplus, Illkirch, France). Transfected cells were cultured in Expi293F Expression Medium at 37 °C, 8% CO_2_, with shaking at 125 rpm for 5 days. Culture supernatants were then harvested by centrifugation, and nanobody–Fc fusion proteins were purified by Protein A affinity chromatography [20] (Ablink Biotech, Chengdu, China).

### 2.4. Determination of Antibody EC_50_

ELISA plates were coated with human or mouse pIgR-ECD (1 μg/mL in PBS) overnight at 4 °C. After blocking with 1% PVA for 2 h and washing with PBS, serial dilutions of purified nanobody-Fc proteins were added and incubated for 2 h at RT. Following PBST washes, HRP-conjugated anti-human Fc antibody was added and incubated for 1 h at RT. After final washes, the signal was developed with TMB substrate, and stopped with 1 M H_3_PO_4_, and absorbance was measured at 450 nm.

### 2.5. Establishment of a Stable MDCK-pIgR Pool

Madin-Darby canine kidney (MDCK) cells were seeded and cultured until reaching approximately 80% confluence. The cells were then transfected with a recombinant plasmid encoding the full-length human pIgR cDNA ORF clone (Sino Biological, Beijing, China) using Lipofectamine 3000 (Thermo Fisher Scientific, Waltham, MA, USA). Following transfection, cells were selected and maintained under stepwise increasing concentrations of hygromycin B (Sigma, MO, USA). Untransfected MDCK cells were cultured in parallel as a control to confirm antibiotic sensitivity. After the control cells were completely eliminated, the transfected cell population was passaged twice more under continued hygromycin selection to establish a stable MDCK-pIgR pool.

### 2.6. Flow Cytometric Analysis of Antibody–Cell Binding

Stably MDCK-pIgR pool cells and wild-type MDCK cells (control) were harvested, counted, and aliquoted at 3 × 10^5^ cells per sample. Cells were washed twice with cold PBS containing 1% fetal bovine serum(FACS buffer, Thermo Fisher Scientific, Waltham, MA, USA), followed by incubation with purified nanobody–Fc fusion proteins (10 μg/mL in FACS buffer) for 45 min at 4 °C. After two additional washes, cells were incubated with Alexa Fluor 488-conjugated anti-human IgG (BioLegend, San Diego, CA, USA, Fc-specific) secondary antibody (diluted 1:200 in FACS buffer) for 45 min at 4 °C in the dark. Following two final washes, cells were resuspended in 300 μL of FACS buffer and immediately analyzed on a flow cytometer (BD FACSCanto™ II, BD Biosciences, San Jose, CA, USA).

### 2.7. Transwell Transcytosis Assay

MDCK-pIgR cells were trypsinized, counted, and resuspended in complete DMEM (Thermo Fisher Scientific, Waltham, MA, USA) supplemented with 10% fetal bovine serum (FBS). Cells (5 × 10^5^) were seeded into the apical chamber of collagen-coated Transwell inserts (BIOFIL, Guangzhou, China, 0.4 μm pore size, PET membrane) in a 12-well plate format. The basolateral chamber was filled with 2 mL, and the apical chamber with 1 mL, of complete medium. Cells were cultured for 5 days at 37 °C under 5% CO_2_ to establish confluent monolayers.

Prior to the transport assay, monolayers were serum-starved for 2 h in DMEM containing 1% FBS. For apical-to-basolateral transcytosis, nanobody–Fc fusion proteins, along with a negative control antibody (41BB-antibody) (Ablink Biotech, Chengdu, China) (15 μg/mL in 1% FBS DMEM), were added to the apical chamber, while fresh 1% FBS DMEM was placed in the basolateral chamber. For basolateral-to-apical transcytosis, antibodies were introduced into the basolateral chamber and fresh medium into the apical chamber. After 48 h of incubation at 37 °C, samples were collected from the opposing chamber—basolateral medium for apical loading, and apical medium for basolateral loading [21]. The concentration of transported nanobody–Fc was subsequently quantified by ELISA.

### 2.8. In Vivo Fluorescence Imaging

Purified nanobodies and a negative control antibody were fluorescently labeled with Dylight™ 800 NHS ester (Thermo Scientific, Rockford, IL, USA) according to the manufacturer’s instructions. Mice were administered the labeled antibodies intranasally at a dose of 5 mg/kg in PBS. Whole-body near-infrared fluorescence (NIRF) imaging was performed and at 2 h, 24 h, and 72 h post-administration using an IVIS^®^ Lumina imaging system (PerkinElmer, Waltham, MA, USA). At 72 h, mice were euthanized, and major organs (lungs, heart, liver, spleen, and kidneys) were harvested for ex vivo fluorescence imaging [22].

### 2.9. Constructing Bispecific Antibodies for the Asthma Mouse Model

The pIgR-targeting nanobodies were genetically fused to the C-terminus of the Reslizumab heavy chain, and the resulting bispecific antibodies were reconstituted and expressed in Expi293F cells following standard protocols.

Thirty-six female BALB/c mice were randomly divided into six groups (*n* = 6 per group): saline control, OVA model control, Reslizumab intraperitoneal (i.p.) 25 mpk, Reslizumab intratracheal (i.t.) 2.5 mpk, Reslizumab-3LTHMP-4 i.t. 3.35 mpk, and Reslizumab-3LTHMP-5 i.t. 3.35 mpk. Mice were sensitized on days 1 and 8 via intraperitoneal injection of 200 μL of OVA sensitization solution containing 50 μg OVA (Sigma, St. Louis, MO, USA) and 1.6 mg alum adjuvant (Thermo Fisher Scientific, Waltham, MA, USA). On days 15, 21, 22, and 23, animals were challenged by intratracheal instillation of 50 μg OVA in 50 μL PBS [23]. Treatments were administered three times (on days 14, 20, and 22) prior to OVA challenge.

On day 24, 24 h after the final OVA challenge, bronchoalveolar lavage fluid (BALF) was collected. Total white blood cell (WBC), eosinophil (EOS), neutrophil (NEU), and lymphocyte (LYMPH) counts in BALF were determined using a Siemens blood (ADVIA) analyzer (Siemens, Erlangen, Germany) with peroxidase staining. IgE levels in serum and BALF were measured using ELISA kits (Elabscience, Wuhan, China) according to the manufacturers’ instructions. Lung tissues were collected, fixed, and subjected to hematoxylin and eosin (H&E) staining and periodic acid–Schiff (PAS) staining. All histological sections were evaluated blindly by two experienced pathologists. The severity of bronchial mucosal mixed cell inflammation was scored on a 5-point scale: 1, mild; 2, mild to moderate; 3, moderate; 4, severe; 5, extremely severe. Goblet cell hyperplasia was quantified by the percentage of PAS-positive cells among total airway epithelial cells, scored as follows: 0, <0.5%; 1, <25%; 2, 25–50%; 3, 50–75%; 4, >75%.

All animal experiments were conducted at Sichuan Greentech Biotechnology Co., Ltd. (Chengdu, China). in accordance with the Guidelines for the Care and Use of Laboratory Animals issued by the National Research Council and were approved by the Institutional Animal Care and Use Committee (IACUC) of Sichuan Greentech Biotechnology Co., Ltd. (Approval No. IAC-B2025122-P-01).

### 2.10. Epitope Analysis by HDX–MS

HDX–MS was performed to map the epitopes recognized by 3LTHMP-4 and 3LTHMP-5. Recombinant human pIgR was incubated with a twofold molar excess of each nanobody in 50 mM HEPES (pH 7.4) (Sigma, MO, USA), 150 mM NaCl, 4 mM TCEP (Thermo Fisher Scientific, Waltham, MA, USA) for 1 h at 4 °C to form stable complexes. For each exchange time point (0, 10, 60, 300, and 900 s), 5 μL of the complex was diluted into 20 μL of D_2_O-based labeling buffer at 4 °C, and hydrogen–deuterium exchange was quenched after the specified time by adding 25 μL of ice-cold quench buffer (4 M guanidine hydrochloride, 1% trifluoroacetic acid). Quenched samples were immediately flash-frozen on dry ice and stored at −80 °C until analysis. HDX–MS analysis was performed using an automated platform (LEAP PAL 3.0, LEAP Technologies, Carrboro, NC, USA), where samples were injected and digested online on an immobilized pepsin column (4 °C, flow rate 120 μL/min). The resulting peptides were trapped and desalted on a C18 trap column, followed by separation over 8 min on a 2.1 mm × 5 cm C18 analytical column (1.9 μm Hypersil Gold, Thermo Fisher Scientific, Waltham, MA, USA) with a linear gradient of 4–40% acetonitrile containing 0.3% formic acid. Throughout digestion and chromatography, the system was maintained at 4 °C. Mass spectrometry data were acquired on an Orbitrap Fusion™ Tribrid™ mass spectrometer (Thermo Fisher Scientific, Waltham, MA, USA) with a resolution of 65,000 at *m*/*z* 400 [24]. The pIgR structural model used for epitope visualization was generated using AlphaFold 3 (https://deepmind.google/science/alphafold/, accessed on 1 March 2026) [25].

### 2.11. Statistical Analysis

Data are presented as mean ± standard error of the mean (SEM). Comparisons between two groups were performed using two-tailed unpaired Student’s *t*-tests. Comparisons among multiple groups were analyzed by one-way analysis of variance (ANOVA) followed by Bonferroni’s post hoc test for multiple comparisons. For histopathological scores (H&E and PAS), the Kruskal–Wallis test followed by Dunn’s post hoc test was applied. Statistical significance was defined as * *p* < 0.05, ** *p* < 0.01, *** *p* < 0.001, **** *p* < 0.0001, and NS, not significant.

## 3. Results

### 3.1. Discovery of Nanobodies

Phage display biopanning against human pIgR-ECD identified four distinct nanobody clones: 3LTHMP-4, 3LTHMP-5, 3LTHMP-18, and 3LTHMP-19. Phage ELISA confirmed strong and specific binding of all selected clones to both human and mouse pIgR-ECD, with negligible reactivity to bovine serum albumin (BSA) (Figure 2B). The selected clones were reformatted as Fc-fusion proteins, expressed in Expi293F cells, and purified by Protein A affinity chromatography. ELISA-based affinity measurements revealed that all four nanobody-Fc proteins bound to human and mouse pIgR-ECD with low-nanomolar EC_50_ values, confirming high affinity and cross-species reactivity (Figure 2C,D).

### 3.2. Antibody Bioactivity Assay

#### 3.2.1. Extracellular Transwell Endocytosis Assay

**Figure 3:**
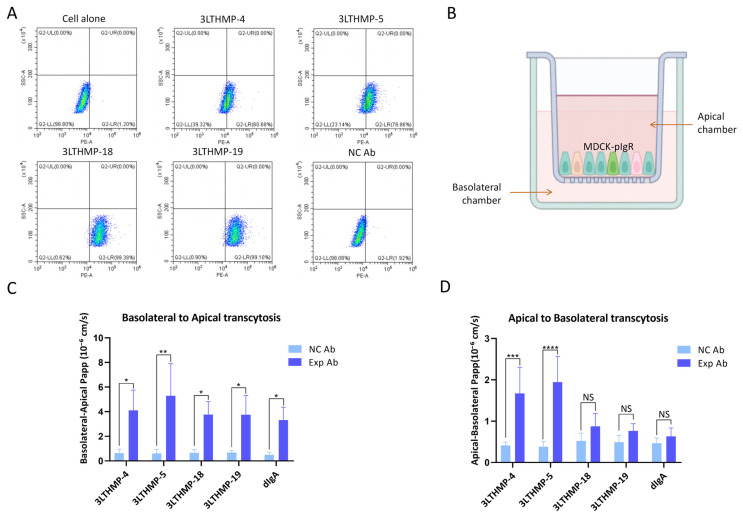
Functional characterization of pIgR-specific nanobodies in a polarized Transwell model. (**A**) Flow cytometric analysis of nanobody–Fc binding to MDCK cells stably expressing pIgR (MDCK-pIgR). (**B**) Schematic diagram of the polarized MDCK-pIgR Transwell model for assessing directional transepithelial transport. Nanobodies and negative control antibody (NC Ab) were added to either the apical or basolateral chamber; translocated antibodies in the opposite chamber were quantified by ELISA after 48 h. (**C**) Quantification of basolateral-to-apical transport after 48 h. The apparent permeability coefficient (Papp, cm/s) was calculated as Papp = (Q/t)/(A × C_0_), where Q = transported amount (ng), t = 48 h = 172,800 s, A = membrane area (4.7 cm^2^), and C_0_ = initial concentration in the donor chamber (15 μg/mL = 15,000 ng/mL). Transport efficiency (%) was calculated as (Q/Q_0_) × 100, with Q_0_ = total initial amount in the donor chamber (30,000 ng for basolateral-to-apical transport because the basolateral volume is 2 mL). Dimeric IgA (dIgA) served as a positive control for pIgR-mediated basolateral-to-apical transcytosis. Data are presented as mean ± SEM (*n* = 3 independent experiments). (**D**) Quantification of apical-to-basolateral transport after 48 h. Papp and transport efficiency were calculated using the same formulas as in (**C**), with Q_0_ = 15,000 ng (apical volume 1 mL, C_0_ = 15 μg/mL). Data are presented as mean ± SEM (*n* = 3 independent experiments). Statistical significance was defined as * *p* < 0.05, ** *p* < 0.01, *** *p* < 0.001, **** *p* < 0.0001; NS, not significant.

Binding of the nanobody–Fc proteins to stably MDCK-pIgR pool cells was assessed by flow cytometry (Figure 3A). All four pIgR-targeting nanobody–Fc constructs exhibited strong binding to MDCK-pIgR cells. In contrast, when using negative control antibody, they hardly bound to the target.

Functional characterization of nanobody transport was conducted using polarized MDCK-pIgR monolayers (Figure 3B). All four nanobody–Fc proteins, along with the natural ligand dIgA, mediated efficient basolateral-to-apical transcytosis (Figure 3C). Notably, only 3LTHMP-4 and 3LTHMP-5 demonstrated substantial apical-to-basolateral transcytosis (Figure 3D). In contrast, 3LTHMP-18 and 3LTHMP-19 showed minimal apical-to-basolateral transport, similar to levels observed with a negative control antibody or dIgA—which undergoes predominantly basolateral-to-apical transport. These results indicate a clear functional divergence: while all nanobodies utilize pIgR for basolateral-to-apical transport, only 3LTHMP-4 and 3LTHMP-5 are capable of engaging the receptor’s apical-to-basolateral transport pathway.

#### 3.2.2. In Vivo Lung Retention and Biodistribution

**Figure 4:**
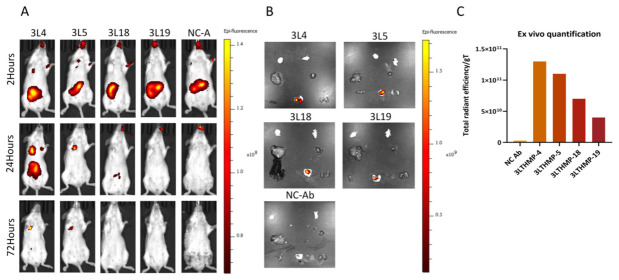
In vivo biodistribution of fluorescently labeled nanobodies in mice. (**A**) In vivo near-infrared fluorescence imaging of mice at 2 h, 24 h and 72 h following intranasal administration of DyLight 800-labeled 3LTHMP-4(3L4), 3LTHMP-5(3L5), 3LTHMP-18(3L18), 3LTHMP-19(3L19) and NC Ab. (**B**) Ex vivo fluorescence imaging of dissected organs at 72 h post-administration. Lungs are highlighted to indicate tissue-specific retention. (**C**) Semiquantitative analysis of ex vivo fluorescence signals. Data are expressed as total radiant efficiency ([p/s]/[μW/cm^2^] per gram of tissue).

To evaluate the in vivo lung retention of nanobodies in mice, mice were intranasally administered DyLight 800-labeled nanobodies. Whole-body near-infrared fluorescence (NIRF) imaging revealed distinct biodistribution patterns among the nanobodies. At 24 h post-administration, 3LTHMP-4 and 3LTHMP-5 exhibited strong and sustained fluorescence localized to the thoracic region, indicating efficient lung retention. In contrast, 3LTHMP-18, 3LTHMP-19, and the NC Ab showed minimal thoracic fluorescence signal. Imaging at 72 h further demonstrated persistent lung-associated fluorescence for 3LTHMP-4 (Figure 4A). 3LTHMP-5 also showed detectable lung signal, though at lower intensity than 3LTHMP-4 and signals for the 3LTHMP-18, 3LTHMP-19 and NC Ab had declined to near-baseline levels. Ex vivo imaging of harvested organs at 72 h confirmed substantial lung-specific retention for 3LTHMP-4 and, to a lesser degree, 3LTHMP-5. Both 3LTHMP-18 and 3LTHMP-19 also exhibited detectable pulmonary retention, but at markedly lower fluorescence intensities compared to 3LTHMP-4 and 3LTHMP-5. Notably, no fluorescence signal was detected in any other major organs for all four nanobodies (Figure 4B,C). In contrast, lung signals for the control nanobody were negligible.

### 3.3. Preparation of Bispecific Antibodies and In Vivo Efficacy Experiments in Mice

#### 3.3.1. Construction of IL5-pIgR Bispecific Antibodies

**Figure 5:**
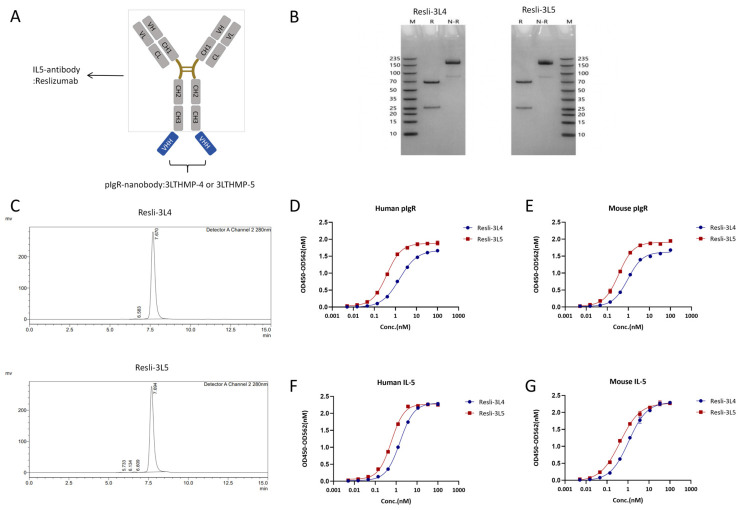
Generation and characterization of bispecific antibodies. (**A**) Schematic representation of the bispecific antibody constructs generated by fusing Reslizumab with 3LTHMP-4 or 3LTHMP-5. (**B**,**C**) Purity analysis of purified bispecific antibodies Reslizumab-3LTHMP-4 (Resli-3L4) and Reslizumab-3LTHMP-5 (Resli-3L5) by SDS-PAGE under reducing conditions (**B**) and size-exclusion chromatography (SEC-HPLC) (**C**). (**D**–**G**) Binding affinity (EC_50_) of Resli-3L4 and Resli-3L5 to (**D**) human pIgR, (**E**) mouse pIgR, (**F**) human IL-5, and (**G**) mouse IL-5.

To validate the therapeutic efficacy of the selected nanobodies as carriers in an asthma model, we constructed two bispecific antibodies by fusing 3LTHMP-4 or 3LTHMP-5 to the anti-IL-5 monoclonal antibody Reslizumab (Figure 5A). Both bispecific antibodies were expressed and purified to high homogeneity, with SDS-PAGE analysis confirming purity > 90% (Figure 5B) and size-exclusion chromatography (SEC-HPLC) demonstrating purity > 99% (Figure 5C). Binding affinity assays revealed that both bispecific antibodies retained potent and specific binding to their respective antigens—human/mouse pIgR and IL-5 (Figure 5D,G).

#### 3.3.2. The Efficacy of Bispecific Antibodies in the Asthma Mouse Model

**Figure 6:**
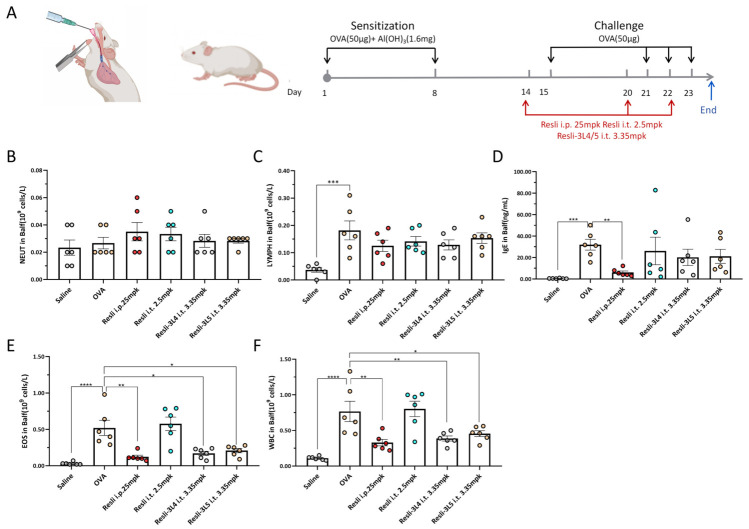
Therapeutic efficacy in an OVA-induced murine asthma model. (**A**) Experimental timeline and dosing schedule for intratracheal (i.t.) and intraperitoneal (i.p.) administration of antibodies in the OVA-induced asthma mouse model. Mice were sensitized and challenged with OVA, followed by treatment with the indicated antibodies. (**B**–**F**) Quantification of inflammatory cell counts and IgE levels in bronchoalveolar lavage fluid (BALF): (**B**) neutrophils, (**C**) lymphocytes, (**D**) IgE, (**E**) eosinophils, and (**F**) total white blood cells. Data are presented as mean ± SEM (*n* = 6 mice per group). Statistical significance was defined as * *p* < 0.05, ** *p* < 0.01, *** *p* < 0.001, **** *p* < 0.0001.

To evaluate the therapeutic efficacy of the bispecific antibodies, we measured inflammatory cell infiltration and IgE levels in bronchoalveolar lavage fluid (Balf) from the OVA-induced asthma mouse model (Figure 6B–F). Compared to the blank control group, the model group exhibited significantly elevated numbers of total white blood cells, eosinophils, and lymphocytes, confirming successful establishment of airway inflammation.

Notably, treatment with Reslizumab alone via intraperitoneal injection (i.p.)—the clinically relevant route—significantly reduced eosinophil and total white blood cell counts, as well as IgE levels, compared to the model group. Remarkably, both bispecific antibodies—Resli-3L4 and Resli-3L5—administered intratracheally (i.t.) at one-tenth the dose of i.p. administration of Reslizumab achieved reductions in eosinophil and total white blood cell counts comparable to those observed with administration of Reslizumab. However, although a downward trend in IgE levels was observed in these groups, the reduction did not reach statistical significance. In contrast, intratracheal administration of unmodified Reslizumab alone at the same low dose (one-tenth of the i.p. administration of Reslizumab dose) failed to produce any significant improvement in these inflammatory parameters.

**Figure 7:**
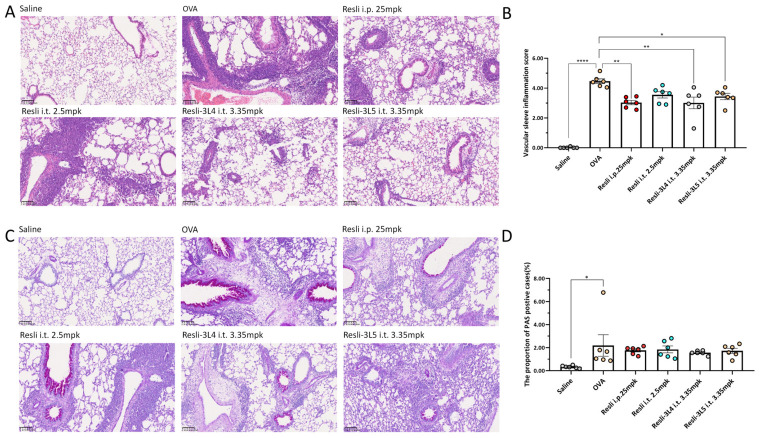
Therapeutic efficacy in an OVA-induced murine asthma model—histopathological analysis. (**A**,**B**) Representative histological sections of lung tissue stained with hematoxylin and eosin (H&E) (**A**) and periodic acid-Schiff (PAS) (**B**). Scale bars, 100 μm. (**C**) Inflammatory scores based on H&E-stained sections, evaluated semi-quantitatively for peribronchial and perivascular inflammation. (**D**) Quantification of goblet cell hyperplasia based on PAS-stained sections, expressed as the percentage of PAS-positive cells among total airway epithelial cells. Data are presented as mean ± SEM (*n* = 6 mice per group). Statistical significance was defined as * *p* < 0.05, ** *p* < 0.01, **** *p* < 0.0001.

Histological analysis further supported these findings. Hematoxylin and eosin (H&E) staining (Figure 7A) and corresponding inflammation scores (Figure 7B) revealed that the i.p. Reslizumab group, as well as both i.t. bispecific antibody groups, markedly attenuated peribronchial and perivascular mixed inflammatory cell infiltration. In contrast, intratracheal administration of Reslizumab alone at the same low dose (one-tenth of the i.p. administration of Reslizumab dose) failed to produce any significant improvement. Periodic acid–Schiff (PAS) staining (Figure 7C) further demonstrated that the i.p. Reslizumab group, as well as both i.t. bispecific antibody groups, reduced goblet cell hyperplasia, although the reduction did not reach statistical significance when quantified as the proportion of PAS-positive cells (Figure 7D).

### 3.4. HDX-MS Combined with Epitope Analysis

**Figure 8:**
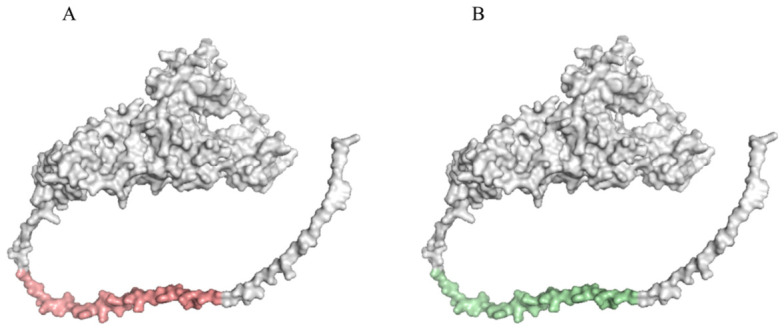
HDX-MS epitope of pIgR-binding nanobodies. Structural representation of the epitopes bound by 3LTHMP-4 and 3LTHMP-5 within the C-terminal stem domain of human pIgR. (**A**) 3LTHMP-4 bound to the pIgR stem domain (structure shown in red); (**B**) 3LTHMP-5 bound to the pIgR stem domain (structure shown in green). The pIgR structure was predicted using AlphaFold 3. The protected region (residues 578–612) is highlighted, corresponding to the sequence ADAAPDEKVLDSGFREIENKAIQDPRLFAEEKAVA. The predicted local distance difference test (pLDDT) confidence scores for this stem zone are below 50, indicating low confidence in the atomic coordinates. However, this structural model is shown for visualization purposes only to illustrate the approximate spatial location of the HDX-MS-defined epitope.

To elucidate the molecular basis for the distinct apical-to-basolateral transcytosis capacity of 3LTHMP-4 and 3LTHMP-5, we performed hydrogen–deuterium exchange mass spectrometry (HDX-MS) to map their binding epitopes on pIgR. HDX-MS analysis revealed that both nanobodies specifically interact with a continuous region spanning residues 578–612 within the C-terminal stem domain of pIgR (Figure 8A,B). The protected peptide sequence was identified as ADAAPDEKVLDSGFREIENKAIQDPRLFAEEKAVA.

## 4. Discussion

The mucosal barrier remains the primary obstacle to non-invasive delivery of large-molecule therapeutics. Monoclonal antibodies targeting IL-5, such as reslizumab and mepolizumab, have demonstrated marked efficacy in treating severe asthma. While effective, these mAbs are limited by their high cost and invasive route of administration. Currently approved mAbs are administered parenterally via intravenous or subcutaneous injections, necessitating the presence of medical personnel [26]. The administration of delivery also presents inherent disadvantages, including low tissue utilization and limited mucosal penetration [27]. For instance, less than 2% of intravenously administered antibodies reach ocular tissues [28], and only approximately 0.2% are recovered in bronchoalveolar lavage (BAL) fluid [29,30]. Moreover, administration of exposure distributes biologics to tissues uninvolved in disease pathology, imposing an unnecessary pharmacological burden and increasing the risk of adverse effects [31]. These limitations highlight the need for inhalation-based delivery, which enables direct airway targeting with reduced dosage, faster onset, and minimized administration of toxicity. However, no inhaled antibodies have received regulatory approval, primarily because airway epithelial cells actively exclude large molecules [32]. The pIgR comprises two main domains: an extracellular SC that binds immunoglobulins, and a membrane-anchored stem zone. Upon ligand dissociation, this stem zone recycles to the basolateral surface via basolateral-to-apical transcytosis—a unique pathway that represents an unexploited gateway for actively ferrying inhaled biologics across the epithelium.

To identify pIgR-targeting nanobodies, we employed phage display on a camelid VHH library [33] and isolated four clones with high affinity for both human and murine pIgR. Based on methodologies reported by Niewoehner et al. [34] for assessing transcytosis, we performed functional screening in a polarized Transwell epithelial model to identify nanobodies with apical-to-basolateral transcytosis capacity. This approach identified 3LTHMP-4 and 3LTHMP-5 as capable of mediating efficient apical-to-basolateral transcytosis, whereas two other clones (3LTHMP-18 and -19) showed minimal activity in vitro. Using the methodology for tracking labeled proteins reported by Lázaro-Ibáñez et al. [22], we performed in vivo pulmonary imaging to assess nanobody distribution. These Transwell results were further confirmed by in vivo imaging, which showed robust accumulation of 3LTHMP-4 and 3LTHMP-5 in lung tissue following intranasal administration. Of note, this in vivo imaging experiment was exploratory, with only one mouse per group and no statistical analysis; the data are shown for trend observation only. Interestingly, despite lacking appreciable in vitro apical-to-basolateral transcytosis activity, both 3LTHMP-18 and 3LTHMP-19 showed detectable lung accumulation exceeding that of the irrelevant control. The reason for this discrepancy remains unclear and warrants further investigation to elucidate the underlying mechanism. Importantly, whole-body imaging revealed that 3LTHMP-4 and 3LTHMP-5 accumulated exclusively in the lungs, with no detectable signal in other organs. Although direct genetic or competitive inhibition of pIgR was not performed, several lines of indirect evidence (specific binding to pIgR-expressing cells, similar kinetics to the natural ligand dIgA, and lack of transport by an irrelevant control nanobody) are consistent with pIgR-dependent transcytosis. However, more definitive experiments, such as pIgR knockdown/knockout or competition with recombinant pIgR, will be required to conclusively establish receptor dependence. Furthermore, this lung-selective distribution underscores their potential as inhalable carriers for respiratory diseases, enabling localized delivery of fused therapeutics while minimizing systemic exposure and off-target toxicity—a critical advantage over systemically administered biologics.

The nanobody format proved particularly well-suited for this application. Its small size (~15 kDa), high stability, and single-gene structure [35,36,37] facilitated construction of bispecific molecules with high expression yields and excellent purity, contrasting favorably with more complex formats such as scFvs, which often suffer from aggregation or low production efficiency. Using this platform, we generated an IL-5-pIgR bispecific antibody by fusing 3LTHMP-4 or 3LTHMP-5 to Reslizumab. In a murine OVA-induced asthma model, intratracheal administration of the bispecific antibody at one-tenth the dose of intraperitoneal Reslizumab achieved reductions in eosinophil and total white blood cell counts comparable to those achieved with administration of Reslizumab. Notably, neither the bispecific antibody nor administration of Reslizumab significantly improved bronchial goblet cell hyperplasia in any treatment group. Given that eosinophilic inflammation is a key driver of airway pathology in asthma [38] and IL-5 is a critical mediator of eosinophil activation and survival [39,40], this lack of effect on goblet cells is consistent with the primary role of IL-5 in eosinophil activation rather than direct goblet cell regulation [41]. These improvements are consistent with those reported by Westerhof et al. [42] in their studies on IL-5-targeted asthma therapies. Indeed, goblet cell hyperplasia is driven by IL-4 and IL-13 in addition to IL-5, and consistent with this, even systemically administered Reslizumab did not significantly reduce goblet cells. However, the reductions in IgE levels and goblet cell hyperplasia did not reach statistical significance. This is likely due to the limited dosing frequency (three intratracheal administrations) and the fact that IgE has a longer half-life and is produced by plasma cells not directly accessible via the airway route. In contrast, i.t. Reslizumab alone at one-tenth the i.p. dose showed no therapeutic effect. These results demonstrate that pIgR-mediated apical-to-basolateral transcytosis enables efficient pulmonary delivery at substantially reduced doses. While these findings provide initial validation of the pIgR-targeted delivery strategy, additional control groups will be needed to fully elucidate the underlying mechanism. Based on the above discussion, we will optimize the dosing frequency and regimen in our future studies to achieve maximal therapeutic benefit across all disease parameters.

To elucidate the structural basis for the differential transcytosis activity observed among our nanobodies, we performed hydrogen–deuterium exchange mass spectrometry (HDX-MS) using methods adapted from Kumar et al. [43] and previously reported protocols. This analysis revealed that both 3LTHMP-4 and 3LTHMP-5 bind a continuous epitope within the pIgR stem zone (residues 578–612), a zone distinct from the secretory component domain engaged by dIgA [44]. This stem zone, which remains membrane-anchored after proteolytic cleavage and naturally recycles to the basolateral side via apical-to-basolateral transcytosis, has been previously associated with this transport direction; our findings are consistent with these models [17,45]. Notably, while previous studies have implicated the stem zone in apical-to-basolateral transcytosis, the precise epitope identified here (residues 578–612) differs from the binding sites previously reported by Gupta et al. [17], which epitoped to residues 462–564 and 607–754. Importantly, binding to this non-competitive epitope preserves normal mucosal immune function—a key therapeutic advantage. It should be noted that HDX-MS epitope mapping was not performed for the non-transcytosing clones 3LTHMP-18 and 3LTHMP-19; therefore, whether they bind to the stem zone or other regions remains unknown. The association between stem-zone targeting and apical-to-basolateral transcytosis is primarily supported by the positive clones, and its generalizability requires further investigation.

Although validated in the pulmonary context, this platform is not anatomically restricted. The pIgR is expressed across multiple epithelial tissues, including the respiratory, gastrointestinal, and reproductive tracts, and the stem zone epitope we identified is conserved at these sites. Consequently, the same nanobody could be formulated as a nasal spray, oral capsule, or topical preparation, enabling non-invasive biologic delivery to diverse mucosal surfaces. Remaining challenges include quantitative characterization of transport kinetics, assessment of species cross-reactivity, and evaluation of efficacy in extra-pulmonary disease models. We also acknowledge that validation in primary human airway epithelial cells or lung-on-a-chip models would further strengthen the translational relevance of this platform, and such studies are planned for future work.

In summary, we identified two pIgR stem zone-specific nanobodies that, when fused to an anti-IL-5 antibody (Reslizumab) and administered intratracheally, effectively ameliorated asthma in a murine model. The current data point to pIgR stem-zone targeting as a promising strategy to improve mucosal delivery. However, direct receptor-dependence studies and epitope mapping of the non-transcytosing clones are still needed to fully understand the underlying mechanism.

## Data Availability

Data is contained within the article. The original contributions presented in this study are included in the article. Further inquiries can be directed to the corresponding authors.
